# Increasing Engagement during Online Learning through the Use of Interactive Slides

**DOI:** 10.1128/jmbe.00117-21

**Published:** 2021-07-30

**Authors:** Nazzy Pakpour, Isabel Souto, Pamela Schaffer

**Affiliations:** a Department of Biological Sciences, California State University East Bay, Hayward, California, USA; b Hayward Unified School District, Hayward, California, USA; c Alameda County Office of Education, Hayward, California, USA

**Keywords:** interactive slides, online learning, distance learning, student engagement

## Abstract

It is difficult in asynchronous online instruction to keep students engaged and motivated. The rapid and unexpected nature of the move to online instruction has meant that the content presented to students has been primarily static and linear. Thus, there is a need for creative pedagogical approaches that re-create some level of the laboratory experience. One economical and accessible approach to building an interactive lab experience is making web-based interactive slides. In the virtual spaces created by this approach, students can explore different modalities of content in a nonlinear and asynchronous manner. We hope that this approach will make the learning process easier and more enjoyable for students while simultaneously making the complex content normally covered in microbiology labs more approachable. In this article we provide detailed instructions for producing web-based interactive slides as well as an example interactive slide that encompasses content that might normally be presented in an introductory microbiology class.

## INTRODUCTION

One of the greatest concerns of college educators, as learning has shifted online, is a lack of student engagement. Many of us have noted how easy it is for students to become bored and unmotivated with remote instruction (1, 2). Engagement refers to the observable and unobservable aspects of student interactions with learning activities and content (3). A wide body of research has demonstrated that engagement increases academic achievement regardless of a student’s ethnic or socioeconomic background (4). Engagement is typically broken down into three components: behavioral, emotional, and cognitive (5, 6). The behavior component is focused on the effort and participation that a student is willing to put toward a particular activity or learning outcome. The emotional component speaks to the interest and connection students feel toward a particular subject matter, while the cognitive component is focused on a student’s investment in learning goals. In-person microbiology laboratory classes not only behaviorally, emotionally, and cognitively engaged students, they also provided a collaborative active learning environment. In the constructivism framework, students understand new ideas and concepts best by forming links to their own prior experiences, a process that requires their active engagement with the material (7). Online instruction struggles to provide that active experience, and most online assignments emphasize and incentivize only the cognitive component of engagement by offering points for the completion of certain tasks.

When laboratories are taught in person, instructors could encourage student participation by assessing their knowledge needs in real-time and guiding their efforts as they navigate the material. Thus, the behavioral aspect of engagement was not difficult to address under these circumstances. However, in asynchronous online instruction, this task shifts from the instructor to the student. How then can we incentivize the behavioral aspect of engagement for students learning online? One solution is to design online content, such as interactive slides, that take a student-centered approach where building knowledge, freedom of choice, and exploitation of a large number of technological modalities can be used (8). Extensive research has shown that students learn best when actively engaged in the learning process (9). Online student engagement behavior has been shown to be the highest, as measured by “views,” for videos, assignments, and materials designed in support of a main online lecture (10). Further, student achievement and engagement in online courses increase when students are offered the flexibility and control to partake of these different materials at their own pace (11–13). Interactive slides can provide students the opportunity to build their content knowledge at their own pace by engaging with a variety of materials and technologies contained within the interactive slide, such as readings, podcasts, videos, websites, traditional homework assignments, and prerecorded lectures. By presenting all the material concurrently, this approach gives students the freedom and control to choose their own learning path. This sense of ownership coupled with the multiple modalities of content presentation has been shown to greatly increase student participation and effort (11–13). When students are exploring material willingly in a playful manner, they are much more intrinsically motivated to learn (14).

**FIG 1:**
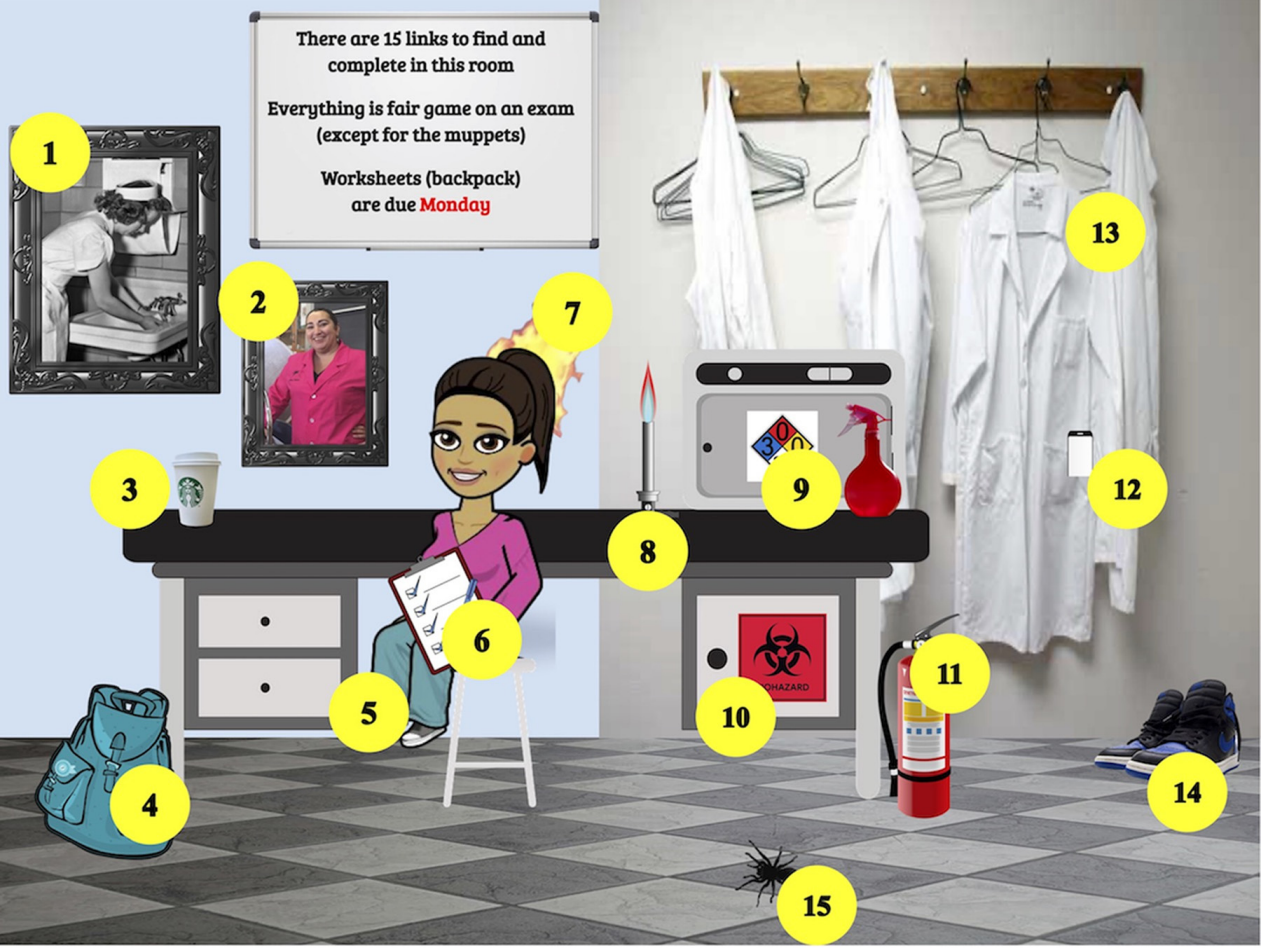
Image of a web-based interactive slide on lab safety with all links labeled. The full interactive slide can be found at https://docs.google.com/presentation/d/1QiGBVkfXhBLtpFMDz6Fnkdh5h6mColo8x2tu4eFLVS0/present?usp=sharing. (1) Framed image of nurse on wall washing hands; reading: history of handwashing (https://drive.google.com/file/d/1lpxeTh3P_3fnBEmuElndRNkWfWAPxSFD/view?usp=sharing). (2) Framed image of instructor; podcast: “White coat feelings” (https://www.npr.org/2016/07/22/486926244/white-coat). (3) Coffee cup; video: general lab safety (https://www.youtube.com/watch?v=dsg1YW6IF4M). (4) Backpack cartoon; Google form: homework assignment (https://docs.google.com/forms/d/e/1FAIpQLSeenofTPDQvLPYlbIz28_8O3ds_013k5Uh8pqF1waB1SkbT1A/viewform?usp=sf_link). (5) Bitmoji image of instructor; video: intro and overview of lab (https://drive.google.com/file/d/1dXmVjPxoTNDMGW0u1S--TNRXxfR3LWif/view?usp=sharing). (6) Clipboard cartoon; reading: overview of lab guidelines (https://drive.google.com/file/d/1sxARPZ5jAv7--zXN5Ikd6b-O78hFyxaf/view). (7) Hair on fire; video: clips of people catching their hair on fire (https://www.youtube.com/watch?v=hjAF8zrsWeo). (8) Bunsen burner cartoon; video: how to use a Bunsen burner (https://www.youtube.com/watch?v=N7ssCM3qM3U). (9) Hazard symbols; website: explanation of hazard symbols (https://www.acs.org/content/acs/en/chemical-safety/basics/nfpa-hazard-identification.html). (10) Biohazard symbol; website: history of biohazard symbol design (https://99percentinvisible.org/article/biohazard-symbol-designed-to-be-memorable-but-meaningless/). (11) Fire extinguisher image; video: Muppets setting things on fire (https://www.youtube.com/watch?v=FnblmZdTbYs). (12) Cell phone image; video: bacteria on cell phones (https://www.youtube.com/watch?v=bKkURz6O3eo). (13) Lab coat image; video: importance of lab coats (https://www.youtube.com/watch?v=a6DrCdjedas). (14) Shoe image; reading: shoe accidents in lab (https://drive.google.com/file/d/1ZHATzlnFrLOKUst_TT4RfmwNyejT95_D/view?usp=sharing). (15) Spider gif; video: instructor being silly (https://drive.google.com/file/d/14OF-cxSSQaMKvuivh8cY8qMK3Ea0bDfY/view?usp=sharing).

It would be difficult to envision an online learning experience that could fully recreate the sheer joy of doing hands-on science in a laboratory. However, we believe that interactive slides can at least capture a small part of the emotional component of engagement for students by providing a means for more self-directed learning. Since students do not know what images on the slide link to which materials, they have to discover each item by exploring. Thus, each student engages with the material in a nonlinear and unique manner similar to that of a game which has been shown to be a highly effective approach (15–17). To further encourage this exploration, fun and silly items, nominally related to the content, are also included in each slide for students to find (Fig. 1, item 7). Finally, to foster a sense of connection and familiarity with the material and their instructor, each room contains a humorous cartoon image of their instructor which links to an introductory video. Humor has been shown to be highly effective in enhancing teaching effectiveness in an online setting (18–20).

**FIG 2:**
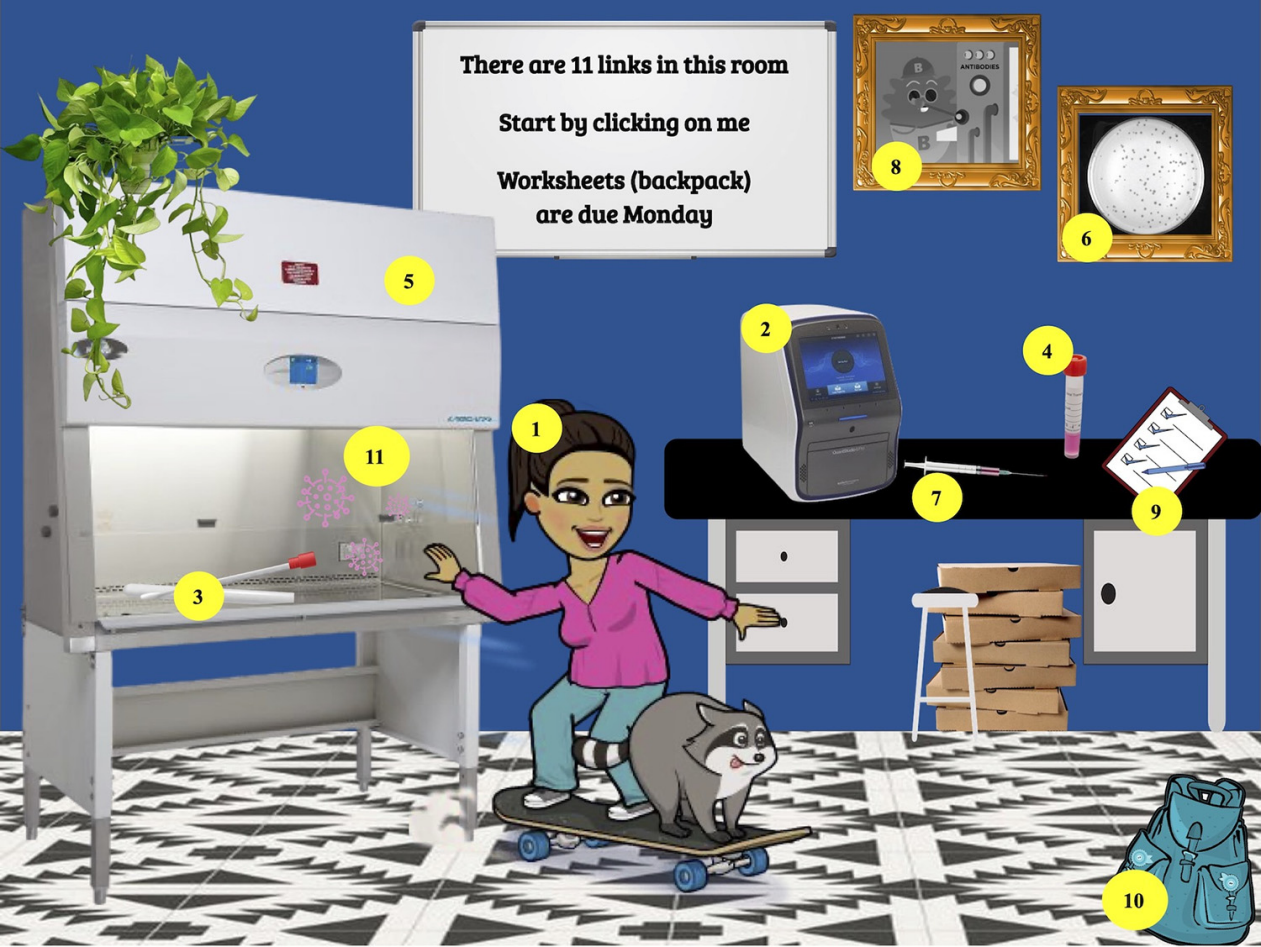
Image of a web-based interactive slide on COVID-19 testing procedures with all links labeled. The full interactive slide can be found at https://docs.google.com/presentation/d/1CifsbQGKfOwkV_F_cB6ypspR8y-gcrJ-ppoi0kGkFFM/edit?usp=sharing. (1) Bitmoji image of instructor; video: intro and overview of lab (https://drive.google.com/file/d/1MpzbS06YudwASs9vpOgyGsLkHiBEdlQk/view?usp=sharing). (2) PCR machine image; video: animation of COVID-19 testing (https://www.youtube.com/watch?v=ThG_02miq-4). (3) Swab image; video: how nasal swabs are collected (https://www.youtube.com/watch?v=DVJNWefmHjE&feature=emb_logo). (4) Tube of viral swab sample; reading: viral transport solution protocol and recipe (https://drive.google.com/file/d/199VQTJEF_C7MXMr8H5lZYixMIm4REJyy/view?usp=sharing). (5) Biological safety cabinet (BSC); video: how to properly use a BSC (https://www.youtube.com/watch?v=96-aZLom340). (6) Plaque assay photo; video: video of plaque assay procedure (https://www.youtube.com/watch?v=er2dwOPwSRo). (7) Syringe; video: how COVID-19 antibody testing works (https://www.youtube.com/watch?v=bYTG7K3YlZ0). (8) B cell animation photo; video: overview of human immune system (https://ed.ted.com/lessons/how-does-the-immune-system-work-emma-bryce). (9) Clipboard; pdf document: student assignment of COVID-19 testing materials (https://drive.google.com/file/d/1j_1JiDINxlmL10-46lcmwy0YDjhXh51J/view?usp=sharing). (10) Backpack; pdf document: student assignment calculating PFU (https://drive.google.com/file/d/1tnFc8TiWdZPSg9mk5xaPvTfvz9B73P1m/view?usp=sharing). (11) Virus gif; video: silly song about COVID-19 safety procedures in school (https://www.youtube.com/watch?time_continue=105&v=oeAN8Xxz0q4&feature=emb_logo).

In a traditional laboratory, students would read a protocol and then perform an experiment. However, it is difficult to simulate a microbiology experiment in an online environment. Therefore, we used interactive slides to have the students take a slightly different approach. Students were asked to watch a video showing how the severe acute respiratory syndrome virus 2 (SARS-CoV-2) genome is detected by PCR (Fig. 2, item 2). Students were then told that they were managers for a new coronavirus disease 2019 (COVID-19) drive-up testing site and they had to write a detailed, step-by-step protocol for the newly hired staff (Fig. 2, item 9). In order to fully understand the procedural video with which they were provided, students had to read or watch additional content provided in the interactive slide, such as the recipe for viral transport medium (Fig. 2, item 4) and how to use a biosafety cabinet (Fig. 2, item 5). Thus, rather than passively reading a standard protocol or watching a video, students had to actively construct their protocol while seeking out the information necessary to do so.

## LEARNING OBJECTIVES

It is difficult to teach safety requirements online for microbiology laboratories previously taught in a hands-on manner. The goal of the interactive slide example presented in Fig. 1 was to familiarize students with the basic safety guidelines of a microbiology laboratory. However, an interactive slide can be used to teach any content and is not a technique specific to the topic of safety. The goal of the interactive slide provided in Fig. 2 was to teach students the basics of the different COVID-19 testing procedures currently in use. In both these examples, students received only the information contained within the interactive slide. After having completed all the materials in the interactive slide in Fig. 1, students should be able to do the following: (i) describe the relevance and history of hand washing and lab coats in preventing infections; (ii) provide evidence for why cell phones should not be used in the lab; (iii) describe accidents that can occur when proper personal protective equipment is not worn; (iv) understand how to operate a Bunsen burner safely; (v) describe the relationship between biohazard groups and lab containment levels; (vi) recognize and define safety icons; (vii) describe basic health and safety measures of a microbiology laboratory; and (viii) identify practices that reduce risk and harm in a microbiology laboratory. After having completed all the materials in the interactive slide in Fig. 2, students should be able to do the following: (i) describe the procedure for collecting and detecting SARS-CoV-2 mRNA using PCR; (ii) name the function of each ingredient in viral transport medium; (iii) list the steps for using a biosafety cabinet properly; (iv) describe the production of antibodies by the human immune system and how COVID-19 antibodies are detected; (v) determine the changes required to turn any space of their choosing into a biosafety level 2 (BSL-2) lab; (vi) summarize the procedure for completing a plaque assay; and (vii) identify the benefits and limitations of PCR, antibody, and plaque assays for the detection of SARS-CoV-2 virus.

## PROCEDURE

The basic premise for making an interactive slide is fairly simple: the objects, images, or gifs on a slide are linked to outside materials such as videos, readings, websites, etc. This can be done in most presentation programs, such as Google slides, PowerPoint or Prezi, by selecting an object on the slide and using the “insert link” tool. The more arduous task is to gather and/or make the materials you want your students to learn. A video tutorial of how to make an interactive slide can be found at https://youtu.be/SrgzEvyyTiY. The image of the instructor was made using the Bitmoji application (https://www.bitmoji.com/) and copied and pasted onto the slide. If images required it, their background was removed using the website https://www.remove.bg/ prior to being added to the slide. Interactive slides are most visually effective if images are layered and arranged in a way that conveys depth. We found that the most dynamic and easy-to-use slides were the ones designed to resemble rooms that had a mixture of real and cartoon/graphic objects in them. We also found that it was important to include objects that we did not link to any outside sources. This facilitated the searching and discovery aspects of student engagement. Basic instructions for how students should engage with the interactive slides were written on the image of a whiteboard contained within the slide. Gifs, such as the moving spider in this slide example, were used judiciously, as they can be visually overwhelming if too many are used at once. All material was also provided on a single pdf document with links for students with accessibility issues or those having trouble navigating the interactive slide.

## CONCLUSION

Interactive slides can provide instructors a way to repurpose existing materials into a new, more dynamic engaging learning modality. These slides can also help instructors leverage the vast array of quality materials that are already available online or are generously being offered by other instructors. We assessed student learning through the completion of the Google form homework assignment linked within the interactive slide. Other alternative forms of assessments instructors might wish to include are written reflective essays or participation in discussion forums based on the content of the interactive slides. This specific activity highlights the ASM curriculum guidelines (21): “Practice safe microbiology, using appropriate protective and emergency procedures” and “Document and report on experimental protocols, results and conclusions.”
